# Impact of antiviral treatment and initiation time on mother-to-child transmission in pregnant women with extremely high HBV DNA loads

**DOI:** 10.1080/21505594.2026.2647479

**Published:** 2026-04-19

**Authors:** Wei Yi, Weihua Cao, Gang Wan, Shiyu Wang, Wen Deng, Ziyu Zhang, Xinxin Li, Yao Xie, Minghui Li

**Affiliations:** a Department of Gynecology and Obstetrics, Beijing Ditan Hospital, Capital Medical University, Beijing, China; b Department of Hepatology Division 2, Beijing Ditan Hospital, Capital Medical University, Beijing, China; c Department of Medical and Biological Statistics, Beijing Ditan Hospital, Capital Medical University, Beijing, China; d HBV Infection, Clinical Cure and Immunology Joint Laboratory for Clinical Medicine, Capital Medical University, Beijing, China; e Department of Hepatology Division 2, Peking University Ditan Teaching Hospital, Beijing, China

**Keywords:** Mother-to-child transmission, chronic HBV infection, HBV DNA load, antiviral therapy

## Abstract

To further reduce mother-to-child transmission (MTCT), this study aims to explore the factors influencing MTCT rate in pregnant women with extremely high HBV DNA loads (HBV DNA ≥ 1 × 10^7^ IU/ml). In this retrospective real-world study, pregnant women with chronic hepatitis B virus (HBV) infection and their infants were analyzed. Pregnant women received antiviral therapy during the second or third trimester based on their HBV DNA loads and personal preferences. Newborns were administered hepatitis B immunoglobulin (HBIG) and hepatitis B vaccine within 6 h after birth, while their HBV infection status was followed up after 7 months. Data from 4,157 pregnant women and 4192 infants were collected. All 35 cases of MTCT occurred in mothers with extremely high HBV DNA loads (1570 mothers and 1588 infants), of which 29 cases were in nonantiviral therapy group, and 6 in antiviral therapy group. Antiviral treatment could markedly reduce MTCT rate (*p* < 0.001). The decrease of HBV DNA loads below 1 × 10^5^ IU/ml or above didn’t impact MTCT rate after antiviral treatment. The average initiating antiviral treatment time among mothers with MTCT was significantly later than mothers without MTCT (*p* = 0.008). In the absence of antiviral treatment, predelivery HBV DNA levels in mothers with MTCT were higher compared to those without MTCT (*p* = 0.001). MTCT mainly occurred in pregnant women with extremely high HBV DNA loads; however, antiviral treatment could reduce MTCT rate. The efficacy of antiviral drugs did not affect the incidence of MTCT, but early initiation of antiviral treatment may contribute to a decrease in its occurrence.

## Introduction

According to data from the World Health Organization (WHO), there are approximately 296 million chronic hepatitis B virus (HBV) infected individuals worldwide, with 1.5 million new cases each year; additionally, around 820,000 people die annually from HBV-related hepatic failure, liver cirrhosis, or liver cancer [1]. The estimated rate of hepatitis B surface antigen (HBsAg) positivity among the general population in China is about 6.1%, with 86 million chronic HBV infected individuals [2]. In China, maternal-to-children transmission (MTCT) is an important way of chronic HBV infection, accounting for 40–50% of new infected individuals [3], mainly occurring in children whose mothers with high HBV DNA load [4]. Therefore, reducing HBV MTCT as much as possible is a crucial strategy for mitigating hepatitis B infection in China and eliminating the harm caused by viral hepatitis by 2030 as outlined by WHO [5].

Timely administration of the hepatitis B vaccine and hepatitis B immunoglobulin (HBIG) into newborns born to mothers have high HBV DNA loads can reduce the MTCT rate to 5–10% [6,7]. Furthermore, incorporating antiviral treatment during pregnancy, such as lamivudine (LAM), telbivudine (LdT), tenofovir disoproxil fumarate (TDF), or tenofovir alafenamide fumarate (TAF), can significantly reduce MTCT rate. These treatments have been shown to be safe and effective for mothers and children [8–13]. However, there is still a relatively low incidence of MTCT despite these antiviral treatments [14], with underlying causes and influencing factors remain unclear.

This is a retrospective real-world study in chronic HBV infected pregnant women who underwent prenatal examination and delivered at Beijing Ditan Hospital. The aim was to explore the possible factors influencing rate of MTCT in pregnant women having high viral level after antiviral treatment, meanwhile, to provide a basis for optimizing existing strategies to prevent MTCT.

## Patients and methods

### Study design and population

**Figure 1.**
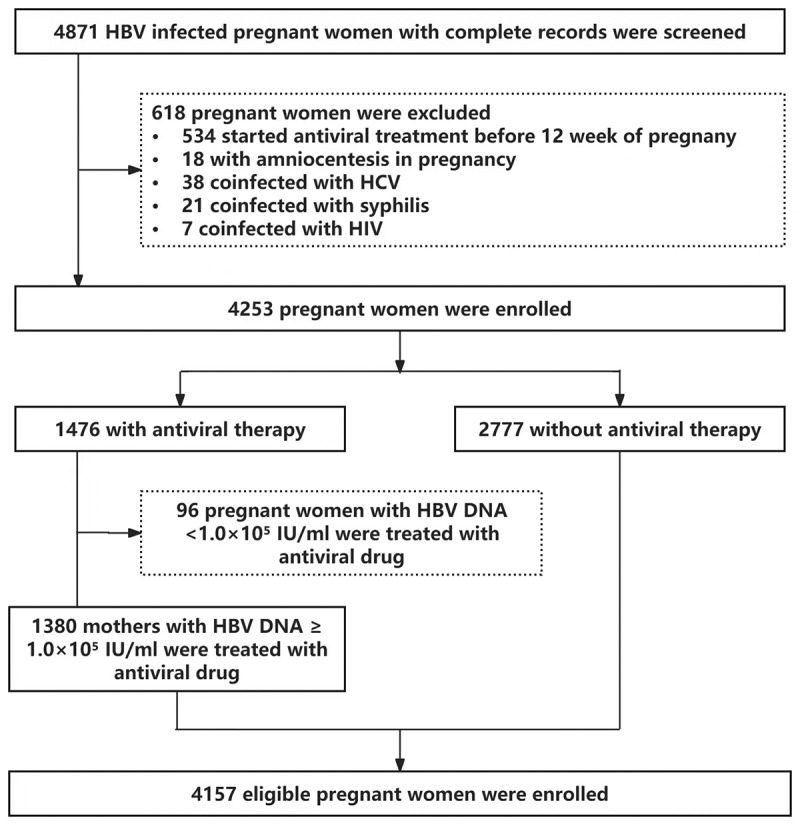
Flow diagram of the patient inclusion procedure. HBsAg, hepatitis B surface antigen; HIV, human immunodeficiency; HCV, hepatitis C virus.

From November 2010 to July 2019, 4157 chronic HBV infected pregnant women who underwent prenatal examination and delivered at Beijing Ditan Hospital were enrolled (Figure 1). Inclusion criteria: (1) pregnant women who have been HBsAg positive for more than 6 months; (2) complete clinical data available for both pregnant women and their infants; (3) HBV serological markers of infants were tested in our hospital 7 months post-birth. Exclusion criteria: (1) women who performed amniocentesis during pregnancy; (2) co-infection with hepatitis C, HIV, TORCH series, syphilis, or other virus; (3) Pregnant women who started antiviral treatment in the early stages of pregnancy or prior to conception were excluded because most of them started antiviral treatment due to abnormal liver function; (4) infants who did not complete the injection of HBIG and hepatitis B vaccine; (5) pregnant women and children lacking complete medical records. The study was approved by the Ethics Committee of Beijing Ditan Hospital (Ethics ID: JDL-2018-043-02) and was exempted from obtaining informed consent form.

The routine prenatal examination process for chronic HBV-infected pregnant women treated at Beijing Ditan Hospital was as follows: prenatal examination every 4 weeks before 28 weeks of pregnancy and every 1–2 weeks after 28 weeks of pregnancy. At second or third trimester of pregnancy, pregnant women with high HBV DNA loads were recommended to take antiviral drugs, including lamivudine, telbivudine, and tenofovir. They decided whether to take medication according to their own wishes and signed an informed consent form. All newborns would be injected with HBIG and hepatitis B vaccine for free within 6 h after birth, followed by one dose of hepatitis B vaccine again in the community hospital at 1 and 6 months after birth, respectively. After completing the last dose of hepatitis B vaccine, they are recommended to detect HBsAg, HBsAb, HBeAg, HBeAb, and HBcAg. If children were infected, HBV DNA would be tested. If HBsAg or HBV DNA of children were positive in venous blood 7–12 months after birth, HBV MTCT was diagnosed [15].

In accordance with the Chinese Guidelines for the Prevention and Treatment of Chronic Hepatitis B (2022 Edition) [16], HBV DNA load more than 2 × 10^5^ IU/ml is defined as high viral level, while chronic HBV infected individuals typically have HBV DNA loads above 2 × 10^7^ IU/ml during the immune tolerance period. In this study, for the convenience of statistical stratification analysis, HBV DNA load more than 1 × 10^5^ IU/ml was defined as high viral levels, and HBV DNA loads above 1 × 10^7^ IU/ml as extremely high viral level.

### Data collection and outcome evaluation

The medical records and laboratory data of pregnant women and their infants were collected from our hospital’s HIS and LIS systems. The information of pregnant women included: age, pregnancy and childbirth history, liver function, HBV DNA, HBV serological markers, antiviral drugs, and the start time of antiviral treatment at the 20–28 weeks of pregnancy (baseline) and before delivery. The information of children included: 5-min Apgar score, birth weight, length, HBV serological markers and HBV DNA loads after 7 months of birth.

The evaluation indicator of this study was MTCT rate of HBV. Positive HBsAg or HBV DNA at 7 months after birth in children was defined as HBV MTCT.

### Laboratory tests

In Beijing Ditan Hospital, all blood tests were performed at the central laboratory. ALT level was tested by a Hitachi 7600 fully automatic biochemical analyzer, and the upper limit of the normal value was 40 U/L (Wako Pure Chemical Industries, Ltd., Osaka, Japan). Serum HBV DNA load was quantified using real-time quantitative PCR by Piji Co., Ltd. (Shenzhen, China) with the limit of detection 100 IU/mL or by Roche (Cobas AmpliPrep/Cobas TaqMan 96) with detection limit 20 IU/ml. Chemiluminescent microparticle immunoassay (Architect i2000 analyzer; Abbott Diagnostics, Abbott Park, IL, USA) was used to detect HBV serological markers.

### Statistics

Categorical variables were summarized as percentages or frequencies, and the difference between two groups were compared by chi-squared or Kruskal–Wallis test. Normal distribution measurement data was described as means ± standard deviations, and the difference between two groups was compared using Student’s *t*-test. Non-normal distribution data was expressed medians and interquartile ranges (Q1, Q3), and Mann–Whitney test was used to compare the difference between two groups. Logistic regression analysis was used to predict influencing factors of HBV MTCT. The Statistical Package for Social Science for Windows, Version 17.0 (SPSS Inc., Chicago, IL, USA) was used to analyze data. All tests were two-tailed with 95% confidence interval, and *p* < 0.05 was defined as with statistical significance.

## Results

### Demographic data

The average age of pregnant women was 32.29 ± 4.06 years old, with average gravidity of 1.94 ± 1.10 and parity of 1.27 ± 0.47. Among 4157 pregnant women, there were 3472 cases of HBV DNA positive, 1964 cases with high HBV DNA levels (HBV DNA ≥ 1 × 10^5^), and 1570 cases with extremely high HBV DNA levels (HBV DNA ≥ 1 × 10^7^). There were 1476 cases who received antiviral treatment, in which 96 cases with low HBV DNA levels were excluded. In the end, there were 1380 cases receiving antiviral treatment during pregnancy were enrolled for analysis.

Among 4192 newborns (including 35 twins), 35 infants had MTCT. There was no statistical difference in baseline ALT, AST, TBil, ALB levels, cesarean section rate, age, gravidity, parity, or gestational week of delivery between pregnant women with (*n* = 35) or without MTCT (*n* = 4122). The baseline HBV DNA load of pregnant women with MTCT was markedly higher than that without MTCT (8.04 ± 0.30 vs. 4.74 ± 3.01 log_10_ IU/ml, *p* < 0.001). The antiviral treatment rate of pregnant women in MTCT group was dramatically lower than that in non-MTCT group (17.1% vs. 33.4%, *p* = 0.042). There was no remarkable difference in gender (male infant rate), birth weight, birth length, and 5-min Apgar score between the two groups of newborns (Table 1).

**Table 1.** Characteristics of pregnant women and their infants.

Values	All population	Non-MTCT group	MTCT group	T or *χ*^2^/*p* value
Mothers	*n* = 4157	*n* = 4122	*n* = 35	
Age (years)	32.29 ± 4.06	32.31 ± 4.06	31.03 ± 4.44	1.842/0.066
No. pregnancies (times)	1.94 ± 1.10	1.94 ± 1.10	2.09 ± 1.15	−0.812/0.417
No. deliveries (times)	1.27 ± 0.47	1.27 ± 0.47	1.43 ± 0.61	−1.521/0.138
Gestational weeks (weeks)	39.05 ± 1.37	39.02 ± 1.40	39.34 ± 1.19	−1.282/0.200
ALT at baseline (U/l)	25.55 ± 47.46	25.54 ± 47.40	19.46 ± 7.12	0.576/0.565
AST at baseline (U/l)	24.05 ± 38.46	24.05 ± 38.41	18.93 ± 4.78	0.598/0.550
TBil at baseline (μmol/l)	7.43 ± 2.55	7.42 ± 2.53	8.27 ± 3.92	−1.466/0.143
ALB at baseline (g/l)	38.61 ± 2.18	38.60 ± 2.18	38.13 ± 2.60	1.003/0.316
HBV DNA at baseline (log_10_ IU/ml)	4.76 ± 3.01	4.74 ± 3.01	8.04 ± 0.30	−47.208/0.000
ALT before delivery (U/l)	17.26 ± 18.83	17.31 ± 18.93	13.99 ± 4.98	1.033/0.302
AST before delivery (U/l)	21.84 ± 17.90	21.90 ± 17.96	18.48 ± 4.98	1.115/0.265
TBil before delivery (μmol/l)	7.92 ± 4.74	7.92 ± 4.74	7.50 ± 1.78	0.525/0.599
ALB before delivery (g/l)	35.21 ± 3.12	35.18 ± 3.14	34.68 ± 3.24	1.006/0.315
HBV DNA before delivery (log_10_ IU/ml)	3.03 ± 2.38	2.99 ± 2.36	7.32 ± 1.41	−17.975/0.000
Treatment rate (*n*, %)	1380	1389(33.4%)	6(17.1%)	4.138/0.042
Cesarean section rate (*n*, %)	1845	1863(44.8%)	16(45.7%)	0.011/0.915
Infants	*N* = 4192	*N* = 4157	*N* = 35	
Rate of boy (*n*, %)	2174	2157(51.9%)	17(48.6%)	0.240/0.887
5-min Apgar score (score)	9.98 ± 0.26	9.98 ± 0.26	10.00 ± 0.00	−0.398/0.691
Birth weight (g)	3359.71 ± 441.48	3353.06 ± 447.95	3285.71 ± 444.34	0.996/0.319
Birth length (cm)	50.07 ± 1.20	50.05 ± 1.23	50.06 ± 1.47	0.053/0.957

Note: MTCT: mother-to-child transmission; ALT: alanine aminotransferase; AST: aspartate aminotransferase; TBIL: total bilirubin; ALB: albumin.

All 35 cases of MTCT occurred in pregnant women having extremely high HBV DNA loads. Among 1570 pregnant women having extremely high HBV DNA loads, a total of 1588 newborns were delivered, and the HBV MTCT rate was 2.20% (35/1588). Among them, the rate of MTCT in pregnant women who received antiviral treatment was 0.51% (6/1179), while it was 7.09% (29/409) in pregnant women without antiviral treatment. Significant difference was shown between the two groups (*χ*^2^ = 61.025, *p* < 0.001), indicating that antiviral therapy during pregnancy could markedly reduce MTCT rate in pregnant women with extremely high HBV DNA levels (Table 1).

### Impact of different baseline HBV DNA loads in pregnant women on MTCT

**Figure 2.**
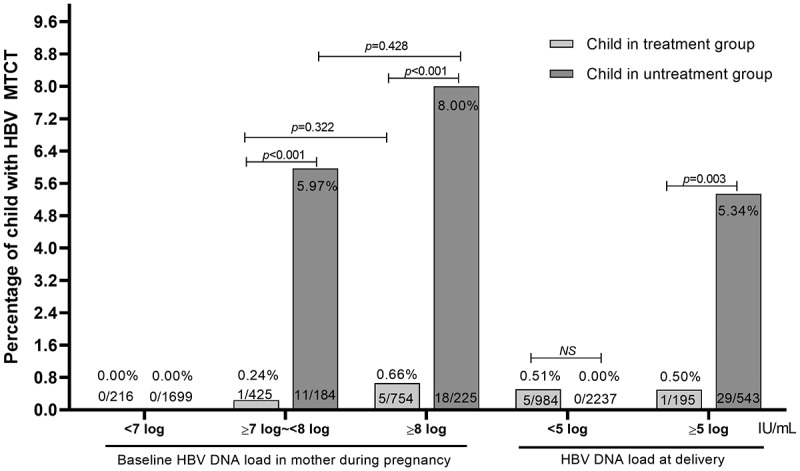
Mother-to-children transmission in pregnant women with different HBV DNA loads. Antiviral treatment significantly reduced MTCT rate in pregnant women having with extremely high HBV DNA loads.

Among 35 cases of MTCT, 29 cases were in women without antiviral treatment in pregnancy and 6 cases in women with antiviral treatment. We conducted a stratified analysis for the impact of different baseline HBV DNA loads of pregnant women on MTCT. The MTCT rate in pregnant women with different baseline levels was shown in Figure 2. When the HBV DNA loads of pregnant women were less than 10^7^ IU/ml, no HBV MTCT occurred after timely injection of HBIG and hepatitis B vaccine in newborns, regardless of antiviral treatment during pregnancy. In pregnant women having baseline HBV DNA load ≥10^8^ IU/ml, the MTCT rate (8.00%, 18/225) in women without antiviral treatment was significantly higher than that (0.66%, 5/754) in women with antiviral treatment (*χ*^2^ = 40.680, *p* < 0.001). In pregnant women having baseline HBV DNA load ≥10^7^ IU/ml to <10^8^ IU/ml, the rate of MTCT (5.97%, 11/184) in women without antiviral treatment was significantly higher than that (0.24%, 1/425) in women with antiviral treatment (*χ*^2^ = 21.925, *p* < 0.001). The results suggested that antiviral treatment could significantly reduce MTCT rate in pregnant women with extremely high HBV DNA loads. In pregnant women receiving antiviral treatment, there was no obvious difference in the MTCT rate between women with HBV DNA level of ≥10^7^–<10^8^ and ≥10^8^ groups (0.24% vs. 0.66%, *χ*^2^ = 0.983, *p* = 0.322), which was similar in pregnant women without antiviral treatment (5.97% vs. 8.00%, *χ*^2^ = 0.628, *p* = 0.428).

In 1179 pregnant women with extremely high HBV DNA level, who underwent antiviral treatment during pregnancy, 132 cases were treated with LAM, 825 cases with LdT, and 222 cases with tenofovir disoproxil fumarate (TDF). Six cases of HBV MTCT were observed, of whom two cases were treated with TDF, and four cases with LdT. The MTCT rate in LAM, LdT, and TDF groups were 0% (0/132), 0.48% (4/825), and 0.90% (2/222), respectively. There was no difference among the three groups (*χ*^2^ = 1.358, *p* = 0.507), suggesting that the three drugs were equally effective in preventing MTCT of HBV.

### Influence of antiviral drug efficacy on MTCT in women with extremely high HBV DNA load

In 1179 pregnant women with extremely high HBV DNA level who underwent antiviral treatment during pregnancy, 6 cases of HBV MTCT were observed. We divided these women into two groups based on the occurrence of HBV MTCT. The baseline HBV DNA levels in the two groups were 8.12 ± 0.24 and 7.99 ± 0.42 log_10_ IU/ml, respectively, which decreased to 4.43 ± 0.70 and 3.87 ± 1.33 log_10_ IU/ml at delivery. Nonparametric testing revealed no significant difference between the two groups at both time points (*Z* = −0.691, *p* = 0.489; *Z* = −1.108, *p* = 0.268). At delivery, one woman (16.67%) in MTCT group (*n* = 6) still had high viral levels (HBV DNA load ≥10^5^ IU/ml) (Table 2), while 188 (16.03%) in no-MTCT group (*n* = 1173) had high viral levels; however, the rates of high viral level in the two groups had no significant difference (Fisher, *p* = 1.000, Table 2). This suggests that the efficacy of antiviral treatment in the two groups is comparable.

**Table 2.** Characteristics of six cases with HBV MTCT.

Cases	1	2	3	4	5	6
Mothers						
HBV DNA at baseline	>1.7 × 10^8^	5.79 × 10^7^	>1.7 × 10^8^	>1.7 × 10^8^	>1.7 × 10^8^	>1.7 × 10^8^
Gestational week of treatment start	30	28	28	29	32	28
HBV DNA at delivery	2.62 × 10^3^	1.68 × 10^4^	4.82 × 10^4^	2.93 × 10^4^	3.68 × 10^5^	1.79 × 10^4^
Delivery week	39	38	38	38	39	39
Delivery mode	V	C	V	C	C	C
Infants						
HBV DNA at birth	<100	8.69 × 10^4^	9.18 × 10^2^	7.02 × 10^3^	3.33 × 10^4^	<100
HBsAg at birth	1.70	>250	1.14	12.60	225.57	0.02
HBV DNA at follow-up	4.02 × 10^8^	4.45 × 10^8^	1.39 × 10^3^	3.50 × 10^8^	8.26 × 10^4^	2.19 × 10^7^

Note: V = vaginal delivery; C = cesarean section; HBsAg: hepatitis B surface antigen.

We further divided pregnant women with extremely high HBV DNA loads into two groups based on the antiviral drugs’ efficacy. The MTCT rate (0.508%, 5/984) for pregnant women who had HBV DNA load decreased to <10^5^ IU/ml at delivery was found to be similar to that for those whose HBV DNA load remained ≥10^5^ IU/mL at delivery (0.513%, 1/195) (*χ*^2^ = 0.000, *p* = 0.993). These results indicate that the efficacy of antiviral therapy does not appear to affect the rate of MTCT of HBV.

### Impact of the time of initiating antiviral treatment on the rate of HBV MTCT in pregnant women with extremely high HBV DNA load

Among 1179 pregnant women with extremely high HBV DNA loads receiving antiviral therapy during pregnancy, 6 infants occurred HBV MTCT during follow-up. The average time of initiating antiviral treatment in the six mothers was 29.17 ± 1.60 weeks, while it was 26.10 ± 5.12 weeks in others. Non-parametric testing revealed a significant difference between the two groups (*z* = −2.661, *p* = 0.008), which indicates that delayed antiviral treatment in pregnant women with extremely high HBV DNA loads may affect the HBV MTCT rate (Table 2).

### Risk factors of MTCT in pregnant women with extremely high HBV DNA loads

Because the risk factors for HBV MTCT may vary in the antiviral treatment and nonantiviral treatment groups, we exposed risk factors in the two groups separately.

There were 409 children born to mothers who had extremely high HBV DNA loads and without antiviral treatment in pregnancy, of which 29 cases of HBV MTCT occurred. The HBV DNA load before delivery in the MTCT group was markedly higher than that in the non-MTCT group (7.91 ± 0.41 vs 7.57 ± 1.07, *p* = 0.001); nevertheless, there was no significant difference in other factors between the two groups (Table 3).

**Table 3.** Analysis of influencing factors of MTCT in pregnant women with extremely high HBV DNA loads.

Varies	Without antiviral therapy			With antiviral therapy		
	Non- MTCT	MTCT	T or χ^2^/*p* value	Non- MTCT	MTCT	T or χ^2^/*p* value
Mothers	*n* = 376	*n* = 29		*n* = 1159	*n* = 6	
Age (year)	31.57 ± 4.03	30.69 ± 4.53	1.118/0.264	31.50 ± 4.01	32.67 ± 3.83	−0.707/0.479
Gravidity (times)	1.96 ± 1.19	1.97 ± 1.05	−0.012/0.990	1.85 ± 1.00	2.67 ± 1.50	−1.322/0.243
Parity (times)	1.27 ± 0.47	1.38 ± 0.56	−1.126/0.261	1.25 ± 0.44	1.67 ± 0.81	−1.252/0.266
Abnormal ALT in pregnancy (n, %)	114(30.3%)	3(10.3%)	2.460/0.177	429(37.0%)	1(16.7%)	2.931/0.087
Positive HBeAg (n, %)	365(97.1%)	29(100%)	0.872/0.350	1130(97.5%)	6(100%)	0.154/0.695
HBV DNA at baseline (log_10_ IU/mL)	7.94 ± 0.44	8.01 ± 0.31	−1.221/0.230	7.99 ± 0.42	8.12 ± 0.24	−0.766/0.444
HBV DNA at delivery (log_10_ IU/mL)	7.57 ± 1.07	7.91 ± 0.41	−3.641/0.001	3.87 ± 1.33	4.43 ± 0.70	−1.038/0.299
Pregnant week at treatment (week)	–	–	–	26.10 ± 5.12	29.17 ± 1.60	−2.661/0.008
Hypertension (*n*, %)	5(1.3%)	0(0.0%)	0.390/0.532	18(1.6%)	0(0.0%)	0.095/0.758
Premature rupture of membranes (*n*, %)	63(16.8%)	6(20.7%)	0.295/0.587	213(18.4%)	1(16.7%)	0.012/0.914
Placenta previa (*n*, %)	2(0.5%)	0(0.00%)	0.155/0.694	4(0.3%)	0(0.00%)	0.021/0.885
Postpartum hemorrhage (*n*, %)	31(8.2%)	2(6.9%)	0.065/0/798	84(7.2%)	0(0.00%)	0.469/0.494
Preterm birth (*n*, %)	22(5.9%)	1(3.4%)	0.290/0.590	54(4.7%)	0(0.00%)	0.293/0.588
Placenta accreta (*n*, %)	1(0.3%)	0(0.0%)	0.077/0.781	4(0.3%)	0(0.00%)	0.021/0.885
IVF-ET (*n*, %)	9(2.4%)	1(3.4%)	0.124/0.724	36(3.1%)	0(0.00%)	0.192/0.661
Caesarean section (*n*, %)	170(45.2%)	12(41.4%)	0.160/0.689	463(39.9%)	4(66.7%)	1.774/0.183
Infants	*n* = 380	*n* = 29		*n* = 1173	*n* = 6	
Male (*n*, %)	203(53.4%)	13(55.2%)	1.009/0.604	625(53.3%)	4(66.7%)	0.435/0.805
1 min Apgar score (score)	9.95 ± 0.30	10.00 ± 0.00	−0.837/0.403	9.95 ± 0.43	10.00 ± 0.00	−0.268/0.789
5 min Apgar score (score)	9.95 ± 0.55	10.00 ± 0.00	−0.462/0.644	9.98 ± 0.20	10.00 ± 0.00	−0.153/0.878
Neonatal weight (g)	3325.84 ± 482.42	3289.65 ± 460.27	0.391/0.696	3325.07 ± 451.54	3266.66 ± 394.54	0.316/0.752
Neonatal height (cm)	49.95 ± 1.51	50.13 ± 1.57	−0.642/0.521	50.03 ± 1.23	49.66 ± 0.81	0.724/0.469

Note: MTCT: mother-to-child transmission; ALT: Alanine aminotransferase; IVF-ET: In Vitro Fertilization and Embryo Transfer.

In 1179 children born to mothers with extremely high HBV DNA loads and with antiviral treatment in pregnancy, 6 cases of HBV MTCT occurred. There was no obvious difference in all possible influencing factors between the MTCT group and non-MTCT group (Table 3).

Based on the above results, in pregnant women with extremely high HBV DNA loads, it is not possible to conduct multivariate logistic analysis on the risk factors of MTCT in the antiviral and non-antiviral treatment group.

## Discussion

The current strategies for preventing HBV MTCT include timely injection with HBIG and hepatitis B vaccine in infants born to HBV infected women and antiviral therapy during pregnancy in pregnant women having high HBV DNA load [16,17]. The MTCT rate is 5–10% if hepatitis B vaccine and HBIG are timely injected after birth [6,7], and it’ll decrease to 0–1.82% if antiviral therapy is initiated during pregnancy in women having high HBV DNA load [9–14]. Our research showed that the incidence of MTCT in pregnant women with high HBV DNA load was 4.92%(29/590) when timely injected with HBIG and hepatitis B vaccine and it would decrease to 0.43% (6/1395) if antiviral drugs were used during pregnancy. Our conclusion is consistent with reports in the literature, and antiviral treatment can significantly reduce HBV MTCT rate.

In our study, we enrolled pregnant women with different HBV DNA level, owing to the timely administration of HBIG and hepatitis B vaccine in newborns after birth, all cases of MTCT just occurred in pregnant women having HBV DNA load ≥10^7^ IU/ml, regardless of whether they underwent antiviral treatment during pregnancy. We defined HBV DNA load ≥10^7^ IU/ml as extremely high HBV DNA level compared to other HBV DNA level. No MTCT occurred in pregnant women with HBV DNA load less than 10^7^ IU/ml. A possible reason is that the sample size of cases with HBV DNA less than 10^7^ IU/ml in this study is small (*n* = 397), so MTCT might not occur due to its low incidence in these women. Our results indicate that clinical doctors should pay more attention to pregnant women having extremely high HBV DNA load and their newborns.

We initially presumed that the efficacy of antiviral therapy during pregnancy might affect the prevention of MTCT in pregnant women with extremely high HBV DNA loads. Our findings indicated that the effectiveness of antiviral therapy was comparable between women with and without HBV MTCT transmission, and there was no significant difference in the MTCT rate among pregnant women with HBV DNA level ≥10^5^ IU/ml compared to those with levels <10^5^ IU/ml prior to delivery, indicating that efficacy of antiviral treatment does not impact MTCT rate. We speculated that MTCT might occur earlier, potentially before the initiation of antiviral therapy in a small subset of individuals. If this speculation is validated, early antiviral treatment might be a viable strategy to further reduce MTCT rates among pregnant women presenting with extremely high HBV DNA load.

Therefore, we analyzed the impact of initiating time of antiviral treatment during pregnancy on MTCT in women with extremely high HBV DNA load, and found that the average initiating time of antiviral treatment in those six pregnant women was 29.17 ± 1.60 weeks in pregnancy. In 1173 pregnant women without MTCT, the average initiating time of antiviral treatment was 26.10 ± 5.12 weeks in pregnancy. Due to the difference in sample size (6 vs. 1173) and the large dispersion of the data in the group without MTCT, nonparametric tests were used after discussion with statistical experts. There was a remarkable difference in the initiating time of antiviral treatment between the two groups, and the initiating time of antiviral treatment in MTCT group was significantly later than that in those without MTCT. This result is consistent with our speculation, suggesting that for pregnant women with extremely high HBV DNA loads, early antiviral therapy can further reduce MTCT rate. However, it is still unclear when antiviral treatment should be advanced to achieve the best health and economic benefits. Contrary to our research, one study reported that initiating time of antiviral therapy at second or third trimester in pregnant women with extremely high HBV DNA level did not have an impact on the effectiveness of preventing MTCT [18]. The inconsistency might be due to the small sample. The meta-analysis included 9 studies including a total of 1502 pregnant women, and the averaged case sample was 166 in each study and only 83 cases in each group [17]. Therefore, no MTCT case might be collected because of its low transmission rate.

Many studies have indicated that the usage of antiviral drugs in early pregnancy or even throughout gestation is safe for mothers and fetuses [19–22]. In 2022, the Guidelines for the Prevention and Treatment of Chronic hepatitis B in China recommended that chronic HBV infected individuals with positive HBV DNA and over 30 years old should receive antiviral treatment [16]. Therefore, we recommend that in pregnant women over 30 with extremely high HBV DNA load should initiate antiviral therapy regardless of gestational age, and it might be beneficial for prevention of maternal diseases and reducing MTCT rate. For pregnant women under 30 years old, it is unclear when antiviral treatment should be initiated, and further prospective research is needed.

Because antiviral treatment is a significant factor affecting the MTCT rate, we decided to explore risk factors affecting MTCT rate in pregnant women with or without antiviral treatment, respectively. In univariate analysis, the predelivery HBV DNA loads in non-MTCT group were remarkably lower than those in MTCT group among pregnant women without antiviral treatment, but no other possible influencing factors were found. This finding suggests that reducing pre-delivery HBV DNA loads through antiviral treatment in these populations can greatly reduce MTCT rate. No high-risk factors affecting MTCT have been found in pregnant women with antiviral treatment. It’s reported that cesarean section could reduce HBV MTCT [23], while we did not get a similar conclusion in this study. We think that cesarean section only plays a role in preventing HBV transmission during labor but not for intrauterine and postpartum HBV transmission. Owing to the current strategies of antiviral therapy in pregnancy and timely injection with HBIG and hepatitis B vaccine after birth in newborns, intrapartum infection is extremely rare. Considering hazards of cesarean section for women, cesarean section is not recommended for the purpose of preventing HBV MTCT. There are studies showing that HBV MTCT was related with quasi species and HBx gene characteristics of mother’s HBV [24,25]. Initiating antiviral treatment as soon as possible for pregnant women with special quasi species or gene characteristics of HBV might be a possible way to reduce MTCT.

This study is a retrospective study with a few of MTCT cases, and the huge difference in sample sizes between MTCT group and no-MTCT group made our research imperfect. Further prospective randomized controlled studies would be warranted. MTCT has been effectively controlled under the current strategy for preventing HBV MTCT. Our research showed that pregnant women having extremely high HBV DNA level constitute a high-risk group for MTCT, and antiviral treatment could significantly reduce this risk. The effectiveness of antiviral treatment may not directly lower the MTCT rate, but initiating such treatment earlier is beneficial for preventing MTCT.

## Supplementary Material

QVIR-2025-0177.R1 - Supplementary material (updated).xls

## Data Availability

Due to the unpublished data and patient privacy protection, we are unable to provide complete raw data, though partial data can be made available at this stage. To ensure transparency, we have provided alternative information, e.g. “detailed analytical procedures,” “representative subsets of the data,” or “processed data with anonymization” in the manuscript and supplementary materials. All results were rigorously validated, and the experimental protocols are thoroughly described to allow reproducibility. Here, we are pleased to provide partial datasets, and these data have been uploaded to the submission system. Data are deposited in National Microbiology Data Center (NMDC) with accession number NMDCX0002157 (https://nmdc.cn/resource/attachment/detail/NMDCX0002157). Meanwhile, necessary information, including name, institution, e-mail, and reasons for needing to view data, are required for a reader or reviewer to apply for access to the data. Nonpublic datasets are available from the first author (Yi Wei at yiwei1215@163.com) upon reasonable request after publication.

## Abbreviations

MTCT, mother-to-child transmission; HBV, hepatitis B virus; HBIG, hepatitis B immunoglobulin; WHO, World Health Organization; LAM, lamivudine; LdT, telbivudine; TDF, tenofovir disoproxil fumarate; TAF, tenofovir alafenamide fumarate.
